# Impact of the Early COVID-19 Pandemic on the Quality of Obstetric Care in a Tertiary Care Center in Karachi, Pakistan

**DOI:** 10.7759/cureus.65401

**Published:** 2024-07-26

**Authors:** Dur-e- Shahwar, Sumaira Naz, Maleeha Naseem, Shamila Saleem, Lumaan Sheikh, Ayesha Malik

**Affiliations:** 1 Obstetrics and Gynaecology, Aga Khan University, Karachi, PAK; 2 Community Medicine, Aga Khan University, Karachi, PAK

**Keywords:** preeclampsia, eclampsia, pph, morbidity, maternal & neonatal outcomes, indicators, quality of care, covid-19

## Abstract

Objective

This study aimed to assess the indirect impact of the COVID-19 pandemic on obstetric quality measures.

Materials and methods

This cross-sectional study was conducted at a private-sector tertiary care hospital in Karachi, Pakistan. Data were collected for specific antenatal, intrapartum, and postpartum care indicators during the initial six months of the COVID-19 phase (March to August 2020) and compared with baseline measures from the preceding six months before the COVID-19 phase (September 2019 to February 2020) using frequencies and percentages.

Results

During COVID-19, there was a 10% reduction (pre-COVID: 1041 and during COVID: 946) in outpatient obstetric volumes and a 65% increase (pre-COVID: 240 and during COVID: 396) in clinic cancellations, indicating a decreased influx of antenatal patients. Teleclinics served 8.3% (1429/18279) of the total obstetric patients during this period.

Marginal decreases were observed in spontaneous vaginal deliveries 1358 (44%) vs 1049 (42.4%) and labor induction rates 818 (26.6%) vs 606 (24.2%). Additionally, there was a slight increase in instrumental deliveries, 121 (3.9%) vs 114 (4.6%) during the COVID phase. However, these changes were not statistically significant. Similarly, no substantial impact was observed on elective and emergency C-sections.

Notably, there were more cases of primary postpartum hemorrhage (PPH) during the COVID-19 phase 36 (1.17%) vs 46 (1.86%), and these changes were statistically significant (p= 0.035). Similar trends were observed for eclampsia (p =0.05) and preeclampsia cases (p-value 0.074). However, other maternal morbidity indicators and intrauterine fetal deaths remained relatively unchanged. NICU admissions increased significantly (p=0.001), while early neonatal deaths remained unaffected. Patient satisfaction rates remained steady for inpatients and improved for outpatients during COVID-19.

Conclusion

The COVID-19 pandemic primarily affected antenatal volumes, neonatal admissions, and maternal morbidity indicators such as PPH, preeclampsia, and eclampsia. Despite the challenges, patient satisfaction and quality care standards were maintained during COVID-19 through new strategies and revised patient care processes.

## Introduction

Pregnancy represents a unique physiological state with altered immune responses, rendering pregnant women more susceptible to infections [1]. Ensuring high-quality, continuous care during the antenatal and postpartum periods is imperative for this vulnerable population [2]. The World Health Organization (WHO) defines maternal and newborn quality of care (QoC) as "the extent to which healthcare services provided to individuals and patient populations improve desired health outcomes, encompassing aspects of access, provision, and experience" [3]. Quality of care can be evaluated using quality indicators that help assess performance against established care standards and attain desired individual and healthcare facility outcomes. These key performance indicators cover input, process, and outcome. Input measures pertain to infrastructure; process measures assess whether the care has been appropriately applied, and outcome measures validate healthcare's ultimate effectiveness and quality [3]. While no unified, comprehensive global list of maternal and neonatal health indicators exists, healthcare facilities routinely monitor specific indicators to assess their performance against standard benchmarks to enhance the quality of care provided [4].

Improving obstetric care quality is crucial for reducing adverse maternal and neonatal outcomes, particularly in low- and middle-income countries (LMICs) [5,6]. However, during pandemics, healthcare facilities face significant challenges in maintaining standards of care. Enormous pressure on healthcare systems with diversion of human resources, alterations in processes, and adjustments in structural aspects to accommodate affected patients disrupts the routine delivery of healthcare services. Furthermore, the utilization of essential services often declines due to the fear of acquiring infection. Additionally, concerns regarding the emerging disease's effect on pregnancy prevent pregnant women from seeking routine antenatal care (ANC) [7]. Data from previous pandemics has revealed that disparities in exposure, susceptibility, and access to treatment have led to poorer obstetric outcomes [8,9]. Similar observations have been highlighted during the Ebola pandemic (2014-2016), and studies from affected areas showed difficulties in the provision of essential maternal healthcare services and a reduction in antenatal visits [10]. One study conducted in Sierra Leone mentioned a 32% reduction in the number of women giving birth and a 60% drop in the cesarean section rates due to low utilization of services, poor health-seeking behaviors, and closure of most health facilities during the outbreak [11].

The situation was similar during the COVID-19 pandemic, and there was a global struggle to overcome the devastating effects of the pandemic. In a systematic review, Gajbhiye et al. [12] evaluated varying effects of COVID-19 and reported an overall higher risk of adverse maternal and neonatal outcomes in LMICs compared to high-income countries. This review conjectured that the affected healthcare during the pandemic could impede progress towards achieving the SDGs (Sustainable Development Goals) related to maternal and child health in LMICs, delaying their attainment. Likewise, in a modeling study by Roberton and collaborators [13], the indirect effects of the COVID-19 pandemic on maternal, neonatal, and reproductive health services (RMNCHs) across 118 LMICs were estimated. The analysis indicated that the disruption of healthcare services could result in a significant increase in maternal and child mortality. It was anticipated that successful efforts to minimize these disruptions and their impact on RMNCHs would lead to a much smaller increase in mortality due to the pandemic.

In South Asia, Pakistan bears one of the highest burdens of maternal (186 deaths per 100,000 live births) and neonatal mortality (41 per 1,000 live births) [14]. The healthcare delivery system in Pakistan encompasses both public and private sectors, organized into a three-tiered structure with primary, secondary, and tertiary healthcare centers. Despite an extensive health infrastructure, healthcare delivery in Pakistan faces several critical challenges, including the high population growth, uneven distribution of health professionals, deficient workforce, insufficient funding, and limited access to quality healthcare services; the COVID-19 pandemic has profoundly affected Pakistan's healthcare systems similar to its effect on other regions of the world [15]. The initial two confirmed cases of COVID-19 in Pakistan were reported on February 26, 2020, in Karachi. As the outbreak began spreading across the country in the third week of March, the government of Pakistan imposed a nationwide lockdown to mitigate virus transmission. While efforts were made to combat COVID-19, maternal health issues received limited attention, resulting in delays in perinatal healthcare services [16].

Throughout the COVID-19 outbreak, our hospital stood at the forefront of providing care to infected patients. It was considered a 'COVID Hospital' due to its available resources, well-trained healthcare staff, comprehensive multidisciplinary facilities, and specialized adult and neonatal intensive care units. Despite immense challenges, several mitigation strategies were implemented in our facility. Healthcare services and processes were adjusted to meet the emerging needs of the increasing COVID-19 burden; Obstetrics and gynecological services were reorganized to focus on COVID-19 patients while minimizing exposure to staff and other patients. Essential inpatient and outpatient obstetric services continued, with early discharges to reduce hospital stays. Teleclinics were introduced for pregnant women and gynecological patients, while elective gynecological surgeries were postponed.

Furthermore, staffing schedules were adjusted to allocate healthcare providers primarily to antenatal clinics, obstetric wards, theatres, and the labor room area. Changes in existing infrastructure allowed for the provision of isolation rooms for pregnant COVID-19 patients and the uninterrupted care of non-COVID-19 pregnant women. Similarly, dedicated theatres were allocated for COVID-19 patients to ensure the smooth operation of obstetric theatres.

Considering pandemics and unexpected events, it becomes imperative for healthcare facilities to conduct assessments of modified processes, updated protocols, and reviews of services to ensure uninterrupted delivery of quality care. We conducted this study to assess the indirect effects of the initial wave of the COVID-19 pandemic on obstetric care by employing quality indicators and contrasting them with established baseline standards. We aimed to scrutinize variations in quality metrics to identify discrepancies and disparities in care processes. This, in turn, could provide insight for targeted interventions to maintain obstetric care standards during pandemics. Such assessments are crucial for healthcare facilities to adapt protocols and ensure uninterrupted, high-quality care during unexpected events like pandemics while upholding quality of care (QoC) standards for future crises.

The aim of the study was to evaluate the impact of COVID-19 on antenatal, intrapartum, and postpartum QoC indicators within the first six months of the pandemic as compared to quality indicators six months before the COVID-19 outbreak at a tertiary care hospital in Karachi, Pakistan.

## Materials and methods

This cross-sectional study was conducted at the Obstetrics & Gynecology Department of a leading tertiary care, private sector hospital (Aga Khan University Hospital, Karachi) with more than 6000 births annually. This facility provides care for low- and high-risk pregnancies.

Data for preselected antenatal, postpartum, and intrapartum quality measures were constantly maintained monthly at the departmental level. Following approval from the ethical review board (ERC) of Aga Khan University Hospital (reference # 2020-4910-11243), a structured proforma was used for data collection of these quality indicators spanning from March to August 2020 (during the COVID pandemic) and September 2019 to February 2020 (before the COVID outbreak).

Quality data confidentiality was rigorously upheld using secondary data, ensuring that patients' identities remained fully protected. This approach involved no direct engagement with individual patients, and all the information collected was meticulously anonymized. The data was exclusively represented in numerical form, devoid of personal identifiers, ensuring no patient-specific or confidential details were disclosed or compromised.

**Figure 1:**
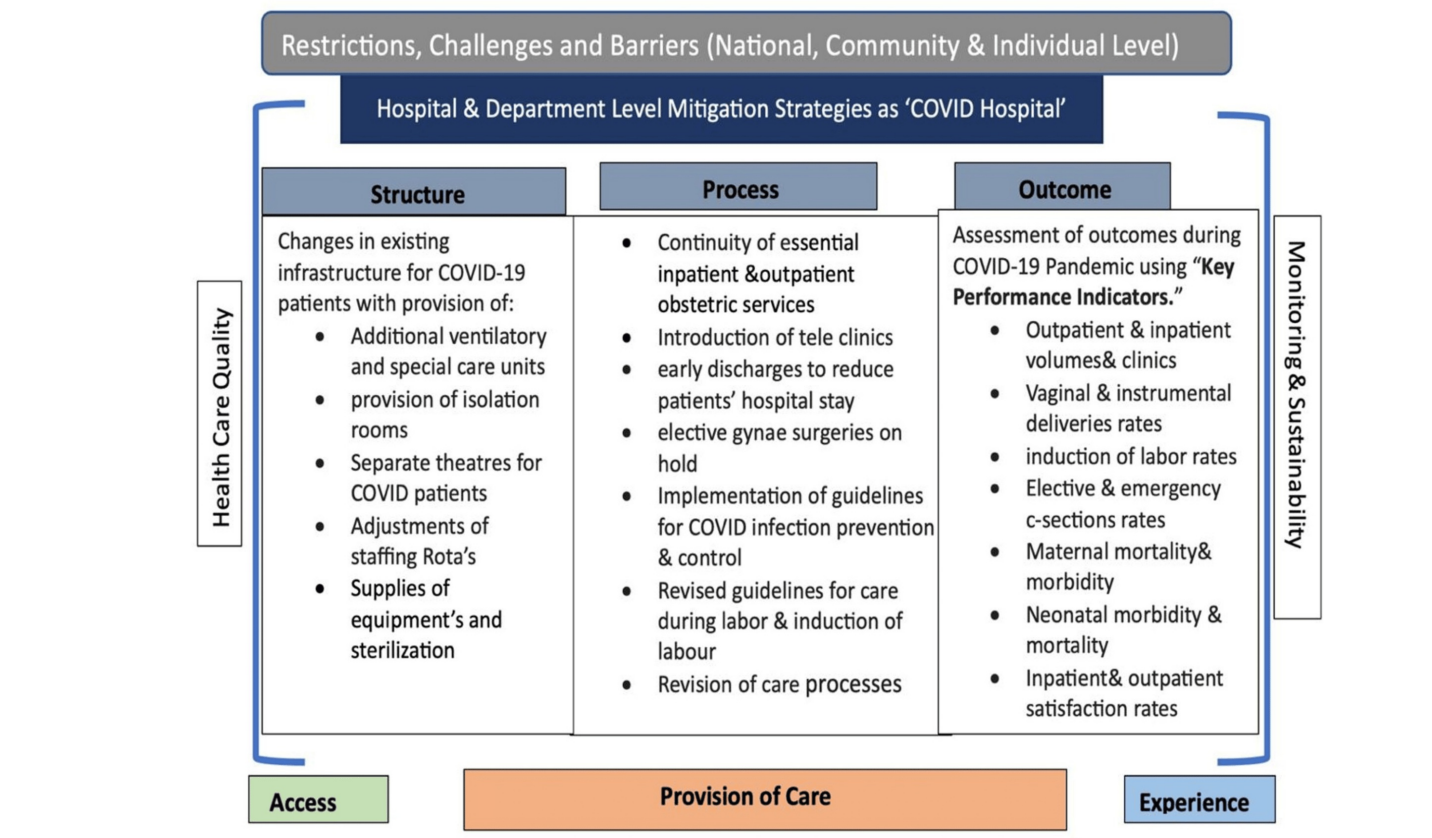
Mitigation strategies to maintain healthcare quality during the early phase of COVID-19. Image credit: This figure was created by the primary author (SN)

These indicators were categorized into process measures (related to antenatal and intrapartum indicators) and outcome measures (about maternal and neonatal morbidity and mortality indicators) to streamline data collection and facilitate comparisons (Figure 1). Antenatal care process measures encompassed metrics such as antenatal clinic volumes, clinic cancellation rates, and the percentage adjustments made for teleclinics. In contrast, intrapartum care indicators included the total number of deliveries, rates of vaginal and instrumental deliveries, induction of labor, and rates of emergency and elective cesarean sections.

Outcome measures comprised maternal morbidity indicators, including postpartum hemorrhage (PPH), obstetric anal sphincter injury (OASI), sepsis, unplanned shifts to the operating room (OR), readmissions within thirty days and maternal mortality among both booked and un-booked cases. Perinatal morbidity and mortality indicators were NICU admissions, intrauterine fetal demise, and early neonatal deaths.

Additionally, we assessed outpatient and inpatient satisfaction rates for both the periods before and after the onset of COVID-19. As part of our routine procedures, the marketing department systematically gathers data for these variables at the hospital level. This includes feedback from outpatient clinic patients and those admitted for inpatient care. The collected data is then shared with each department for review and subsequent actions. Inpatient scores encompass all the touchpoints within a patient's journey when admitted for any procedure or surgery. The survey consists of patient satisfaction and experience statements that patients or their primary attendants are asked to rate on a 5-point Likert scale, where one represents 'strongly disagree,' and five corresponds to 'strongly agree.' These surveys are conducted via telephone within two weeks of the patient's discharge. Patients are also asked to rate their overall satisfaction with their experience during their stay at the facility. Similarly, for outpatient satisfaction scores, patients are requested to assess various attributes using a 5-point Likert scale, and they are also invited to share their overall experience at the clinics, typically within two weeks of their consultation. The scores for each attribute are subsequently calculated by averaging the ratings and converting them into percentages.

All statistical analyses were performed using IBM SPSS Statistics for Windows, Version 19 (Released 2010; IBM Corp., Armonk, New York, United States). Antenatal, intrapartum, and outcome indicators were reported in frequencies and percentages. The chi-square or Fisher exact test was applied to compare the intrapartum and outcome indicators proportion between two COVID periods. p<0.05 was considered as significant. 

## Results

In this study, the outpatient obstetric volumes exhibited a 23818 vs 18675 (21.5%) decline within the first six months of the pandemic compared to quality indicators six months before the COVID-19 outbreak. There was a 1041 vs 946 (10%) reduction in antenatal clinics, whereas clinic cancellations increased to 240 vs 396 (65%). Out of total obstetric volumes, 8.2% of patients (1492/18279) were catered in teleclinics (Table 1). We observed a marginal reduction in booked cases during the six months of the pandemic (97.5% vs. 96.7%; p = 0.075). These patients were registered for antenatal care and delivery at the facility and had at least two visits. Un-booked patients were the self-referred or referred patients from other small clinics or healthcare setups. These cases increased by nearly 12%, while self-referral patients decreased by 18%.

**Table 1:** Comparison of antenatal indicators before and during the COVID-19 pandemic. The asterisk showed teleclinics

Antenatal Indicators	Pre COVID (Sep2019-Feb 2020)	During COVID (March- Aug 2020)	Percent (Increase or decrease)
Obstetric volumes	23818	18675	21.5% ¯
Antenatal clinics	1041	946	10% ¯
Clinic cancellations	240	396	65% ­
*Teleclinic volumes	-	1429/18279 (8.2%)	-

A total of 5538 deliveries were performed between September 2019 and August 2020, of which 2469 (44.6%) were observed during the COVID-19 pandemic, and 3069 (55.4%) occurred before. The overall number of deliveries decreased by 18.6% during this period. The rate of spontaneous vaginal delivery, instrument delivery, induction of labor, and C-section was not statistically significant before and during the COVID-19 pandemic period, as shown in Table 2. 

**Table 2:** Comparison of intrapartum indicators before and during COVID-19. The asterisk shows that the indicator rate is mentioned as a percentage (%) in the table and a p-value of < 0.05.

Intrapartum Indicators*	Pre -COVID (Sep 2019-Feb 2020) n=3069	During COVID (March-Aug 2020) n=2469	*p-value
Spontaneous vaginal delivery rate	1358(44.2%)	1049(42.4%)	0.189
Instrumental delivery rate	121(3.9%)	114(4.6%)	0.216
Induction of labor	818(26.6%)	606(24.5%)	0.075
Total C-section rate	1590(52%)	1306(52.8%)	0.421
Cesarean section rate			
Elective C-section rate	685(6.3%)	596(7.0%)	0.169
Emergency C-section rate	905(18.3%)	710(16.7%)	

The rate of PPH was significantly higher during the COVID phase than before the pandemic (1.17% vs. 1.86%; p = 0.035). Similarly, rates of eclampsia and preeclampsia showed trends toward significance. Regarding neonatal outcomes, NICU admissions were significantly higher during the COVID phase than before COVID-19 (9.4% vs. 7%; p = 0.001). Preterm cases were not significantly different between these two relative phases (80.37% vs. 81.11%, p = 0.842). The rate of intrauterine deaths (64% vs. 40.62%; p = 0.039) and the number of neonatal deaths (72.73% vs. 45.45%; p = 0.016) were significantly higher in booked cases during the COVID-19 pandemic phase as compared to before the pandemic phase. However, the overall effect on intrauterine death, stillbirth, and neonatal deaths was not significant, as reported in Table 3. There were more un-booked maternal mortalities, whereas other maternal morbidity indicators, including readmission within thirty days, birth injuries, and intrauterine deaths, were not affected. In-patient satisfaction was maintained during the COVID era (91.30% vs. 91%; p=0.69), whereas outpatient satisfaction showed a 2.4% improvement compared to the COVID phase (91.50% vs. 93.90%, p=0.0007).

**Table 3:** Maternal and neonatal morbidity and mortality indicators before and during the COVID-19 pandemic. The asterisk showed that the p-value is significant if < 0.05. OASI: Obstetric anal sphincter injury

Outcome Indicators*	Pre -COVID (Sep 2019-Feb 2020) n=3069	During COVID (March-Aug 2020) n=2469	*p-value
Maternal morbidity			
Post-partum hemorrhage	36(1.1%)	46(1.8%)	0.035
Preeclampsia	19(0.6%)	26(1.05%)	0.074
Eclampsia	1(0.03%)	5(0.20%)	0.056
OASI 3^rd^ & 4th degree perineal tears	12(0.3%)	10 (0.4%)	0.934
Readmission within 30 days	6(0.19%)	2(0.08)	0.264
Maternal death	3(0.10%)	4(0.16%)	0.503
Intra-uterine death/still birth	32(1.04%)	25(1.01%)	0.912
Booked	13(40.6%)	16(64%)	0.039
Un-booked	19(59.3%)	9(36%)	
Birth injury	5(0.1%)	6(0.2%)	0.504
NICU admissions	214(7%)	233(9.4%)	0.001
Term	42(19.6%)	44(18.8%)	0.842
Preterm	172(80.3%)	189(81.1%)	
Neonatal deaths	44(20.0%)	33(14.1%)	0.759
Booked	20(45.4%)	24(72.7%)	0.016
Un-booked	24(54.5%)	9(27.2%)	

## Discussion

Our study delved into the indirect repercussions of the first wave of COVID-19 on obstetric quality of care within a private tertiary care center in Pakistan. We observed the effects of this emerging pandemic on obstetric volumes, antenatal clinic attendance, neonatal admissions, and maternal morbidity indicators, including PPH, preeclampsia, and eclampsia. Despite these challenges, the overall standards of care remained consistent, and patient satisfaction levels were upheld throughout COVID-19.

During the outbreak, our tertiary care hospital was one of the leading referral centers for COVID-19 patients. In the face of a mounting caseload of infected individuals, the provision of essential services for non-COVID-19 obstetric patients was ensured, aligning with the government’s directive to continue ANC, labor, and delivery services while implementing stringent infection prevention and control measures; we observed a slight increase in un-booked referred cases. This trend could be attributed to the closure of small maternity centers, concerns surrounding home deliveries, and a prevailing desire for safe childbirth in a health setting during challenging times [17].

We also observed a 10% reduction in antenatal volumes, which may be linked to fear of virus transmission, quarantine measures, transportation restrictions, government-enforced lockdowns, and a lack of antenatal education. Our findings align with the study of Pant et al., who reported a similar impact of the COVID-19 pandemic on antenatal care services, including reduced utilization, clinic hours, and the number of visitors in maternity clinics, to mitigate the risk of infection and reduce the number of ANC visits, teleconsultation services were integrated with in-person care for non-COVID pregnant patients in early pregnancy and for COVID-19-positive patients during their isolation period [18]. In contrast to Semaan et al., who reported a slight overall increase in labor inductions during the first wave, we observed a comparatively reduced number of labor inductions. This was possibly secondary to reduced social inductions due to pregnant patients and their families avoiding unnecessary hospital exposure [19]. We also observed reduced rates of total number of deliveries and spontaneous vaginal deliveries with a slight increase in instrumental delivery rates before and during the pandemic. However, these observed trends were statistically insignificant. Similarly, we identified no significant changes in elective and emergency C-section rates before and during the COVID-19 phase. Our findings align with a study focusing on cesarean section rates in New York City hospitals during the early COVID wave, which also reported no substantial differences in percentages between COVID and non-COVID patients (31.3% versus 39.9%) [20]. Nonetheless, these results could be due to reporting the direct effects of COVID-19 infection on maternal condition, more aggressive obstetric management, changes in quality of care, and variations in intrapartum fetal heart rate monitoring during labor in different healthcare settings. However, it’s worth noting that our study focused on the indirect impact of COVID-19 on quality standards over a period, irrespective of patients` COVID-19 status. Moreover, in our hospital setup, the partial halt of gynecological services led to a heightened focus on obstetric care by obstetric and anesthesia teams, contributing to adaptability and the preservation of care processes. Thus, the sustainability of practices reflected maintaining consistent standards during the COVID-19 phase.

We observed more patients with primary PPH during the COVID phase, and this trend demonstrated statistical significance (p-value 0.035). This association has also been reported in a case series involving two patients, and other studies have mentioned an association between coagulopathy, thrombocytopenia, and worsening COVID-19 infection [21]. This might explain the increased risk of PPH in infective patients. We also noted an increase in instrumental deliveries, which could be another contributing factor to the increased frequency of postpartum hemorrhage. As our study is a cross-sectional secondary data analysis, further prospective studies are recommended to establish any causal relationship between PPH and COVID-19.

We also observed trends toward significance for eclampsia and preeclampsia. Our findings align with a recent study, “The PAN COVID study” by Mullins et al. [22], which reported an increased incidence of eclampsia with COVID infection in the United Kingdom (2.7 per 10,000 births) and suggested an association between infection and a more severe form of the placental disease.

Overall, maternal deaths remained statistically unchanged, but there were more un-booked maternal mortalities during the first phase of COVID-19. These findings are consistent with a study by Mwobobia that showed higher stillbirths and maternal deaths were reported from LMICs in association with COVID-related restriction measures. Our study did not find any significant effect on intrauterine deaths or other maternal morbidity indicators, including OASI and readmission within thirty days, and these findings are in line with the findings of a multicenter prospective study [23].

We did not observe any change in the proportion of preterm babies transferred to the NICU. The overall increase in the number of neonates shifted to the NICU was primarily attributed to logistic considerations related to isolating neonates born to mothers with suspected or confirmed COVID-19. During the initial phase, there was no dedicated ward available for keeping neonates of mothers with proven or suspected COVID-19. Consequently, all neonates born to mothers in this category were shifted to the NICU for isolation purposes, not because they were medically unwell. We observed an increase in neonatal mortality; however, the overall effect of COVID-19 on neonatal deaths did not reach statistical significance. Our findings were consistent with the systematic reviews on the impact of COVID-19 on maternal, perinatal, and neonatal outcomes [24].

During the first wave of COVID-19, inpatient satisfaction was maintained, while outpatient satisfaction showed further improvement compared to the pre-COVID phase. Our findings regarding patient satisfaction were higher compared to another study, which reported satisfaction proportions of less than 60% [25].

During the COVID pandemic, a study by Liu et al. [26], which evaluated health service delivery satisfaction for public and private sectors in Lahore, Pakistan, mentioned higher patient satisfaction with improved services, better care, and a patient-centered approach. Local and global literature also indicates better patient satisfaction and more focused care in private-sector hospitals. In our study, improved patient satisfaction could be attributed to the prompt mitigation measures taken at the hospital level, as the first COVID-19 case in Pakistan was reported from our facility. Previous experiences dealing with emergencies and infectious outbreaks may have helped our facility adapt early to pandemic-related changes. Despite workforce challenges, service adaptations, and the burden of COVID cases, the sustainability of outcome indicators reflects efforts to ensure the continuity of ANC, the preservation of care processes, and the delivery of intrapartum services.

Furthermore, the introduction of teleclinic services could have improved outpatient satisfaction. There was also a reduction in neonatal deaths, and the overall NICU census was much lower compared to the pre-COVID era, possibly resulting in improved one-on-one care for patients.

Strengths and limitations

Our study represented the individual private sector facility-based data that assessed the impact of the COVID-19 pandemic on obstetric QoC during the first wave only. The represented changes could reflect a more focused effect of lockdown in healthcare delivery rather than large-scale causal association or mapping of the pandemic impact in various settings. Hence, duration and generalizability were the limitations. Furthermore, we used secondary data that was collected for quality indicator analysis. Individual patients’ direct information was not available. However, our study shared the unique concept of QoC indicators as impact and performance assessment measures. Our findings reinforced the importance of considering maternal and child health in future pandemic responses. In this context, prospective studies for maternal and child QoC evaluation can better represent the overall impact of pandemics.

## Conclusions

During the early phase of the pandemic, a reduction in obstetric volumes and antenatal clinics, along with affected indicators of PPH, eclampsia, and neonatal admissions, reflected the impact of COVID-19 on patients, providers, and system levels. However, patient satisfaction and overall quality care standards were maintained during the COVID-19 pandemic, indicating our health system's resilience and adaptability despite unexpected health challenges. Our health care facility is a tertiary care hospital that is well equipped with ICU, NICU, and special care facilities, adequate staffing, and expertise, and where the guidelines and care processes are followed. These factors helped the system to combat the enormous pressures during the COVID-19 pandemic and helped provision of essential services with less pronounced effects on specific intrapartum indicators. However, this is different for the health care systems in other areas of Pakistan and LMICs where lack of facilities, equipment, and expertise are related to high maternal and neonatal mortality even before COVID-19. Based on observations, our study highlights the importance of quality indicators for objectively assessing low-resource setups' performance to mitigate strategies for improvement during pandemics, situations of immense burden, and pressure on healthcare systems.
